# Supplementary material for: Determinants of incomplete immunization among 12–23 months old children in Ethiopia: A multilevel analysis
Source: PLoS One. 2025 Apr 29;20(4):e0321947. doi: 10.1371/journal.pone.0321947 (PMC12040096; doi:10.1371/journal.pone.0321947)
Supplement: Attachment — Submitted filename: plos one review on immunization.docx [file pone.0321947.s001.docx]

Thanks! for PLOS ONE editor office for giving me a chance to review this research article titled “Determinants of incomplete immunization among 12-23 months old children Ethiopia:

A Multilevel analysis”

**General comment to authors:**

This work is valid from a scientific standpoint, technically sound, and original to the literature.

On the other hand, the work has received some minor editing, and other corrections.

• Despite Ethiopia's lack of recent EDHS, it would be beneficial to get the most recent data on the factors that contributing to incomplete immunization currently, as we are now in 2025,those factors in 2019 were might be reduced or eliminated.

**Abstract part**

Line 35-36: 3 or more antenatal care (ANC) visits, you mean it is significant factor to have incomplete immunization? It isn’t clear.

**Conclusion part**

Line 40: Since the aforementioned notion runs counter to your finding on inadequate ANC, may we conclude that three or more ANC follow-ups were insufficient/inadequate? And better if you add recommendation too.

**Main body of the Manuscript**

**Materials and methods part**

Line 107-109: Despite the fact that the prior administrative system is crucial to this investigation, it is best to describe the current one as well.

Line 184-185: “if VIF is greater than 10 considered as there is multi-collinearity” What is the numerical result of this study? Write it if possible.

Line 228,238, 253, 292, 299,318: “(Error! Reference source not found.)” is not clear. If you have missed references here insert it please.

**Result and Discussion Part**

It is well-structured, discussed, and scientifically sound.

Lastly, since this poll was conducted at the national level, it would be preferable if you include recommendation as country level.
