# Supplementary material for: Determinants of incomplete immunization among 12–23 months old children in Ethiopia: A multilevel analysis
Source: PLoS One. 2025 Apr 29;20(4):e0321947. doi: 10.1371/journal.pone.0321947 (PMC12040096; doi:10.1371/journal.pone.0321947)
Supplement: Attachment — Submitted filename: Rebutal letter.docx [file pone.0321947.s004.docx]

Date: 11 March 2025

**To: PLOS ONE**

**Subject: Submission of Revised Manuscript**

Dear Editor and Reviewers,

We are grateful for the commitment, feedbacks and reviews on our manuscript **#** **PONE-D-24-57676R1**, titled **"** **Determinants of incomplete immunization among 12-23 months old children in Ethiopia: A Multilevel analysis**". Your detailed and careful comments are really substantial to enhance the quality of the manuscript.

In accordance with the reviewers comments and the journal requirements, we tried to address all the raised issues diligently. In light of the constructive feedbacks, we have meticulously revised and updated the manuscript. Furthermore, an English language expert has reviewed the manuscript to correct any grammatical inaccuracies and we did not cite any retracted articles in this manuscript.

We have keen interest to publish the manuscript in your reputable journal, **PLOS ONE,** to reach a relevant audience and influence policy changes aimed at eradicating vaccine preventable disease in Ethiopia and other similar settings.

Once again, we would like to appreciate the editor and reviewers for their time and constructive feedback. We have provided responses to all comments point by point below.

Best regards,

Yawkal Tsega

Corresponding author

On behalf of the authors

Email:yawkaltsega@gmail.com

Mobile: +251933559351

**Response to Reviewer 2 comments**

**Comment 1:** Authors addressed all comments appropriately. I am recommending publishing this manuscript.

**Authors’ response**: Dear Reviewer 2, we are highly thankful for your positive evaluation of our paper. Your detailed revision has been highly instrumental to improve the quality of the paper.

**Response to Reviewer 3 comments**

**Comment 5:** Line 35-36: 3 or more antenatal care (ANC) visits, you mean it is significant factor to have incomplete immunization? It isn’t clear. I got you in this case it cannot contradict with existing knowledge, or disparities biological plausibility. But your abstract result on both line 35 and on first page of PDF, still says,"≥3 antenatal care visits (AOR: 2.32; 95%CI: 1.43-3.75)" which contradicts with your response, please use the sign "≤" rather than ≥ according to your response.

**Authors’ response**: Dear Reviewer 3, we appreciate your suggestions and for bringing this point to our attention. We revised it as your recommendation in the revised the manuscript.
